# 19 Influence of Co-morbid Modifiable Risk Factors on Adipose-Derived Stem Cell Paracrine Factors in Burn Patients

**DOI:** 10.1093/jbcr/irae036.019

**Published:** 2024-04-17

**Authors:** Jared M Robinson, Jenna Dennis, Sophia Trinh, Cameron Fontenot, Herb A Phelan, III, Jeffrey E Carter, Jonathan E Schoen, Alison A Smith

**Affiliations:** Louisiana State University Health Sciences Center, University Medical Center (LSU Health), Louisiana, New Orleans, LA; Louisiana State University Health Sciences Center, University Medical Center (LSU Health), Louisiana, New Orleans, LA; Louisiana State University Health Sciences Center, University Medical Center (LSU Health), Louisiana, New Orleans, LA; Louisiana State University Health Sciences Center, University Medical Center (LSU Health), Louisiana, New Orleans, LA; Louisiana State University Health Sciences Center, University Medical Center (LSU Health), Louisiana, New Orleans, LA; Louisiana State University Health Sciences Center, University Medical Center (LSU Health), Louisiana, New Orleans, LA; Louisiana State University Health Sciences Center, University Medical Center (LSU Health), Louisiana, New Orleans, LA; Louisiana State University Health Sciences Center, University Medical Center (LSU Health), Louisiana, New Orleans, LA

## Abstract

**Introduction:**

Delayed post-operative wound healing contributes to complications resulting in significant morbidity, mortality and healthcare costs. Co-morbid conditions can act as modifiable risk factors (MRFs) in post-operative wound healing, and their influence has previously been described. However, comparisons of subcellular impacts are less well understood. Adipose-derived stem cells (ADSCs) stimulate a repair response in burned tissue using paracrine signaling factors to alter the surrounding cellular environment. In this pilot study, we tested the hypothesis that the paracrine factor profiles secreted by ADSCs isolated from damaged adipose tissue at the time of burn injury would be altered with differing combinations of MRFs.

**Methods:**

Adipose tissue was collected from adult patients (N=15) with severe burn injuries (>20% total body surface area) and non-severe burn injuries (N=2) at the index operation. ADSCs were extracted and cultured in vitro. Supernatants were harvested 30 hours after plating and used for cytokine determinations by Multiplex assay. Fluorescence activated single cell sorting () confirmed their phenotype with markers CD 90, CD 166, and CD 73. Analysis of variance (ANOVA) and Tukey’s postestimation were performed to compare cohorts with and without comorbid substance use disorder (S-MRF), cardiovascular disease (V-MRF), nutritional deficiency (N-MRF), or a combination of concurrent MRFs (SVN-, SV-, SN-, and VN-MRF).

**Results:**

Profiles from the VN-MRF cohort showed a significant difference (p < 0.05) in IL-6 concentrations from V-, N-, SVN-, and SV-MRF cohorts and IL-8 from S-, V-, N-, SV-, and SVN-MRF cohorts. Profiles from the SV-MRF cohort showed a significant difference (p < 0.05) in IFN-y concentrations from S-, V-, and N-MRF cohorts and IL-17 from V-MRF cohorts. No significant differences in supernatant concentrations of IL-1 beta, IL-4, IL-10, IL-13, TGF-a, TNF-a, FGF-2, MCP-1, VEGF, or MRF were observed (p>0.05).

**Conclusions:**

This study suggests ADSC paracrine factor expression at the time of a burn injury may vary with different combinations of comorbidities and potentially in demographics with a higher burden of chronic illness.

**Applicability of Research to Practice:**

Future studies are needed to examine how modifiable risk factors impact wound healing and post-operative outcomes in burn patients.

